# Eradication of *Pseudomonas aeruginosa* Biofilms Using the Combination of *n*-butanolic *Cyclamen coum* Extract and Ciprofloxacin

**DOI:** 10.5812/jjm.14358

**Published:** 2014-02-01

**Authors:** Morvarid Shafiei, Ahya Abdi Ali, Fereshteh Shahcheraghi, Azra Saboora, Kambiz Akbari Noghabi

**Affiliations:** 1 Department of Biology, Faculty of Science, Alzahra University, Tehran, IR Iran; 2 Department of Bacteriology, Pasteur Institute of Iran, Tehran, IR Iran; 3 Department of Molecular Genetics, National Institute of Genetic Engineering and Biotechnology, Tehran, IR Iran

**Keywords:** *Pseudomonas aeruginosa*, Biofilm, *Cyclamen coum*

## Abstract

**Background:**

Biofilm formation is a major pathogenic factor in different bacteria such as *Pseudomonas aeruginosa*. A number of studies have reported that bacterial biofilms show different levels of antibiotic resistance. In order to re-sensitize the bacterial biofilms to antibiotics, biofilms should be dispersed.

**Objectives:**

In this study, the effect of *n*-butanolic *Cyclamen coum* extract in combination with ciprofloxacin was examined on one, three and five day old *P. aeruginosa* biofilms. The synergistic effect of *n*-butanolic *C. coum* extract and ciprofloxacin towards dispersing pre-established *P. aeruginosa* biofilms was also studied.

**Materials and Methods:**

The ability of biofilm formation by six different *P. aeruginosa* strains was confirmed by microtiter plate method and PCR assay for the *cupA* gene. The extraction of *C. coum* tubers was achieved by fractionation method using different solvents. The minimum inhibitory concentration (MIC) of *n*-butanolic *C. coum* extract and ciprofloxacin against planktonic cells was evaluated using agar well diffusion and microdilution methods. The microdilution chequerboard method was used to determine the fractional biofilm eradication concentration index (FBCI), when the combination of *n*-butanolic *C. coum* extract and ciprofloxacin were used against *P. aeruginosa* biofilms.

**Results:**

The ability of biofilm formation by *P. aeruginosa* strains was quantitatively confirmed. The PCR method confirmed the existence of *cup A* gene (172 bp) in all studied strains. Saponin content of the *n*-butanolic *C. coum* extract was 156 µg/mL. The extract revealed antibacterial activity against planktonic cells of *P. aeruginosa* strains. The results showed that one and three day old biofilms are affected by either ciprofloxacin or *n*-butanolic *C. coum* extract. However, *n*-butanolic *C. coum* extract in combination with ciprofloxacin was significantly more effective against *P. aeruginosa* biofilms.

**Conclusions:**

Using *n*-butanolic *C. coum* extract in combination with ciprofloxacin offers a novel strategy to control biofilm-based infections caused by *P. aeruginosa*.

## 1. Background

Biofilms are organized communities of sessile bacteria that grow on different surfaces. Bacteria can develop biofilms on submerged surfaces such as natural aquatic systems, living tissues, tooth surfaces, indwelling medical devices and implants. One of the most medically important biofilm-forming species is *Pseudomonas aeruginosa*, which is commonly associated with lung infections in patients with cystic fibrosis. Biofilm formation by *P. aeruginosa* occurs through several stages including, attachment to the surface by surface flagella and proteins (such as cup A), followed by division to form microcolonies, and lastly maturation including expression of matrix polymers. Sessile bacteria display phenotypes and possess properties that are considerably different from planktonic cells (1). They are much more resistant to antimicrobial treatments and as a result, biofilms can cause particularly devastating chronic infections or facilitate life-threatening nosocomial infections in short time courses.

 Considering many biofilm-related problems, it is clear that finding an effective and safe medication with biofilm dispersal properties is one of the important issue in clinical microbiology and infectious disease (2). Once a biofilm has been dispersed, the planktonic bacteria become rapidly susceptible to the conventional antibiotics. Therefore, various treatments such as different vaccines, antibiotics and interferon gamma have been studied for biofilm dispersal properties. However, active constituents such as plant extracts may be a better source for biofilm dispersion (3).

Plant-based medicines and derivatives not only have effective roles in the treatment of biofilm-based diseases but also, have fewer side effects in comparison to synthetic medicines. The use of plant extracts and phytochemicals, with antimicrobial and biofilm dispersal properties, can be of great significance in therapeutic treatments. In the last few years, a number of studies have been conducted in different countries to prove such capacities. Many plants have been used because of the compounds they synthesize in their secondary metabolism.

*Cyclamen coum* is a perennial understory herb from the *Myrsinaceae* family, which is endemic to the forests of North of Iran. According to the Ahmadbeigi et al study, the tubers of *C. coum* contain large amounts of saponins and they can be extracted and accumulated in organic phase using different solvents such as petroleum ether, 70% ethanol and *n*-buthanol (4). 

Saponins are a diverse group of compounds widely distributed in the plant kingdom, which are characterized by their structure containing a triterpene or steroid aglycone and one or more sugar chains. Saponins as commercially significant compounds, have expanding applications in food, cosmetics, and pharmaceutical industries due to their anticancer, antioxidant, antihypertensive, antibacterial, antifungal and antiviral activities (5).

## 2. Objectives 

The aim of the present study was to elucidate the dispersal property of *n*-butanolic *C. coum *extract in combination with ciprofloxacin toward pre-formed *P. aeruginosa* biofilms.

## 3. Materials and Methods

### 3.1. Bacterial Strains

Four clinical strains which had been used in previous studies, P.A 214, P.A 25, P.A 29 and P.A 50, along with two standard strains, *P. aeruginosa* PAO1 and *P. aeruginosa* ATCC 8821M, were used in this study (6). Frozen stocks were prepared in trypticase soy broth containing 20% glycerol. Strains were sub-cultured weekly on nutrient agar and stored at 4°C.

### 3.2. Biofilm Formation Assay 

A modified microtiter plate test was used to determine the biofilm formation. An overnight culture of each strain was grown in trypticase soy agar plus 0.2% glucose (Merck, Germany) for 20 hours at 37°C. The bacterial suspensions were prepared in trypticase soy broth plus 0.2% glucose medium according to 0.5 McFarland turbidity standard as measured by absorbance (0.08 - 0.1 at 625 nm) using a spectrophotometer (Schimadzu, model UV-120-01, Japan). Bacterial suspensions (200 μL) were aliquoted in to a 96-well polystyrene microtiter plate (Sigma Aldrich, St. Louis, Missouri, USA) and incubated at 37°C for 24 hours without agitation.

After 24 hours of incubation at 37°C, the content of each well was aspirated, and each well was washed with sterile physiological saline to remove all non-adherent cells. The attached bacteria were fixed with absolute methanol for 10 minutes. The plates were stained using crystal violet (1%W/V). Excess stain was washed off and the plates were rinsed with tap water. The bound dye was resolubilized with 200 µL of glacial acetic acid (33%, v/v). The OD of each well was measured at 570 nm using an ELISA reader. Uninoculated wells containing media served as blanks. A 3-grade scale was used to evaluate the ability of biofilm formation: Negative: ODs < 0.500; Positive: ODs 0.500-1.500; Strongly positive: ODs > 1.500. All tests were carried out in triplicates (7).

### 3.3. Detection of cup A Gene

DNA was prepared by boiling lysis of the bacteria. The *cupA *gene sequence, which was provided from the *Pseudomonas *Genome Database (http://www.pseudomonas.com), utilized for designing primer sequences using the primer3 version 7 software. 

The primers (Forward -5`- CTACCGCTATTCCACCGAAG-3` and reverse-5`-AGGAGCCGGAAAGATAGAGG-3`) were used in this study. 

PCR: amplification of targeted DNA was carried out in 25 μL reaction volumes, each containing 2 mM MgCl_2_, 250 μM dNTP, 0.4 μM (each) primer, 1 U of Taq polymerase (Invitrogen, Carlsbad, Calif.), and 10 μL of whole-cell bacterial lysate, and adjusted to 25 μL by adding high-performance liquid chromatography-grade H_2_O.The amplification was carried out in Thermo Cycler (Merck Company, Germany). After an initial denaturation for 5 minutes at 94°C, 40 cycles were completed, each consisted of 40 seconds at 95°C, 45 seconds at 59.4ºC (annealing temperature), and 60 seconds at 72°C. A final extension of 7 minutes at 68°C was applied. The PCR products were analyzed on 1% agarose gels, visualized under UV illumination and photographed. 

### 3.4. Saponin Extraction

Tubers of *C. coum* were collected from the Golestan Forest (Golestan Province in Iran). Freshly collected tubers of *C. coum* were dried in the shade, sliced into small pieces and pulverized using a mechanical grinder. The dried powder (100 g) was successively extracted using petroleum ether (300 mL) and 70% ethanol (300 mL) using the Soxhlet apparatus (Bio Thechnics, India). After 24 hours, the extract was fractionated by *n*-buthanol in to two phases. Lower phase (*n*-butanolic) which contained *cyclamen* saponins was dried by a Rotavapor (Buchi Flawil, Switzerland). The solid precipitate was dissolved in dimethyl-sulphoxide (DMSO), taking in to account that the maximum concentration of DMSO in the test solution should not exceed one percent (4). 

### 3.5. Determination of Total Saponin Content

Total saponin content was determined by the vanillin-sulfuric acid method. *n*-butanolic *C. coum* extract was mixed with vanillin (8%, w/v) and sulfuric acid (72%, w/v). The mixture was incubated at 60˚C for 10 minutes followed by cooling in an ice water bath for 15 minutes. The absorbance was measured at 538 nm. All determinations were carried out in triplicates. Saponin (1 mg/mL) (Merck, Germany) was used to determine the calibration curve (8).

### 3.6. Determination of Antibacterial Activity of the Extract

The agar well diffusion method was used for evaluation of antibacterial activity of *n*-butanolic *C. coum* extract. About 100 µL of microbial suspension (1×10^8^ CFU/mL) was mixed with molten Muller Hinton agar medium and poured into sterile Petri dishes. Nine wells were made in each 15 cm plate. Thereafter, different concentrations of *n*-butanolic *C. coum* extract were added in three wells of each plate. Negative and positive controls were 100 µL of diluted PBS and ciprofloxacin, respectively. After overnight incubation at 37°C the inhibition zones were measured.

### 3.7. Antibacterial Activity of Ciprofloxacin against P. aeruginosa Strains

Minimum inhibitory concentrations (MICs) and minimum bactericidal concentrations (MBCs) of ciprofloxacin (Sigma-Aldrich) were determined for six *P. aeruginosa* strains using the broth microdilution method according to the procedures outlined by the Clinical Laboratory Standards Institute (CLSI) guidelines.

### 3.8. Biofilm Dispersion Assay

In this study, the effects of *n*-butanolic *C. coum *extract with or without ciprofloxacin on one, three and five day old biofilms were determined. As mentioned in section 3.2, biofilms were established in microtiter plates (Sigma Aldrich, St. Louis, Missouri, USA). After 24, 72 or 120 hours, the media were discarded from the wells and the plates were washed thoroughly with PBS (pH = 7.4), to remove unattached cells. Thereafter, checkerboard arrangements of *n*-butanolic *C. coum* extract and ciprofloxacin were made and added to the pre- formed biofilms. In the checkerboard technique, two drugs are placed in microtiter wells with drug concentrations equal to, above and below their MIC. In our study, the chosen ciprofloxacin concentrations were from 0.25 to 64 µg/mL and selected *n*-butanolic *C. coum* extract concentrations were from 50 to 500 µg/mL. 

Medium (trypticase soy broth plus 0.2% glucose) alone was added to a subset of the wells to serve as the control. Each microtiter plate contained three wells as sterility controls, three wells as growth controls, nine wells for different concentrations of *n*-butanolic *C. coum* extract and antibiotic alone and the combination of the *n*-butanolic *C. coum* extract and antibiotic. The plates were incubated for 24 hours at 37°C and stained with crystal violet (CV). The fractional biofilm eradication concentration index (FBECs) was determined for the combination of *n*-butanolic *C. coum* extract and antibiotic as follows:

FBECA = MBECA(c) / MBECA(a) ,

FBECB = MBECB(c) / MBECB(a)

Σ FBEC = FBECA + FBECB

Where MBEC denotes minimum biofilm eradication concentration, subscripts A and B denote antibiotic and *n*-butanolic *C. coum* extract, subscripts in parentheses, (c) and (a) denote the activity measurements in combination and alone, respectively. The sum of both FBECs was used to classify the combination of *n*-butanolic extract and ciprofloxacin as follows: Synergistic (ΣFBEC ≤ 0.5), partially synergistic (0.5 < ΣFBEC ≤ 1), indifferent (1 < ΣFBEC ≤ 4), or antagonistic (ΣFBEC > 4) (10).

### 3.9. Statistical Analysis 

Data analysis was performed using Excel 2010 software. The results of the experiment were expressed as mean ± standard deviations (SD) for three replicates in each test. The significance of all the data was tested with the Student’s t-test (P < 0.05) using Excel 2010 software.

## 4. Results 

**Figure 1.**
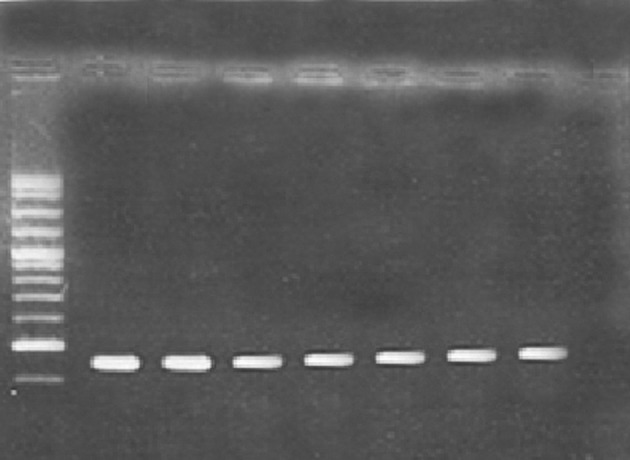
PCR Results From left to right: Marker (M), Positive control (1), PAO1 (2), 8821M (3), PA214 (4), PA25 (5), PA29 (6), PA50 (7) and Negative control (8). The PCR product is 172bp.

Microtitre plate and PCR methods were used to determine the biofilm forming capabilities of *P. aeruginosa* strains. The results revealed that *P. aeruginosa* strains PA25 and PA29 were weakly positive (0.5 – 0.7 OD), *P. aeruginosa* PAO1 and PA 50 were moderately positive (1.3-1.5 OD) and *P. aeruginosa* strains 8821M and PA214 (1.7 -1.8 OD) were strongly positive in biofilm formation. It seems that, these differences are due to the diversity of matrix materials. According to Ghafoor et al. study, highly mucoid *P. aeruginosa* (such as 8821M and PA214) produces biofilms with alginate origin matrix whereas, non-mucoid *P. aeruginosa* (such as PAO1) produces biofilms with Psl, eDNA and proteins origin matrix (3). As shown in Figure 1, all strains examined in this study, contained *cupA* gene with a resulting fragment of 172 bp. 

The type of organic solvents had a profound effect on artifact formation as a result of extraction and improved the rates of isolation of the saponins from cyclamen tubers. According to standard curve, the saponin content of the *n*-butanolic *C. coum* extract was 156 µg/mL. The *n*-butanolic *C. coum* extract revealed antibacterial activity against planktonic *P. aeruginosa*. The data pertaining to the antimicrobial potential of the *n*-butanolic *C. coum* extract and ciprofloxacin are presented in Table 1.

**Figure 2.**
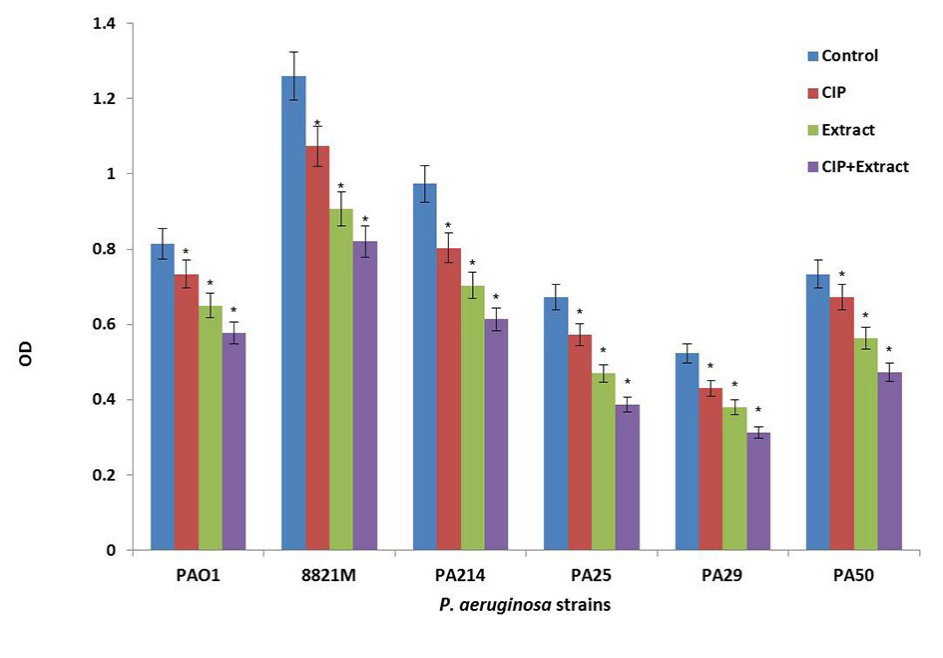
The Effect of Ciprofloxacin (CIP), *n*-butanolic *C. coum* Extract (Extract) and the Combination of Ciprofloxacin and *n*-butanolic *C. coum *Extract (CIP+extract) on one day old Biofilm of Six *P. aeruginosa* Strains Bars represent the mean OD570 values, and error bars represent the standard deviations. Blue bars, control samples treated with TSB medium alone; red bars, samples treated with ciprofloxacin (CIP); green bars represent biofilms treated with *n*-butanolic *C. coum* extract (Extract); purple bars represent biofilms treated with the combination of ciprofloxacin and *n*-butanolic *C. coum *extract (CIP+Extract). Statistical analysis was done using the Student’s t-test the P value < 0.05 was considered as significant (noted with *).

Figure 2 shows the effect of ciprofloxacin (CIP) or *n*-butanolic *C. coum* extract as well as their combination (CIP + plant extract) on one day old biofilm of six strains of *P. aeruginosa*. The antibiotic (at 4×MIC) and *n*-butanolic *C. coum* extract (at 74 µg/mL) affected one day old biofilm mass but their combination had substantial effect on *P. aeruginosa* biofilm mass (P ˂ 0.05). 

**Figure 3.**
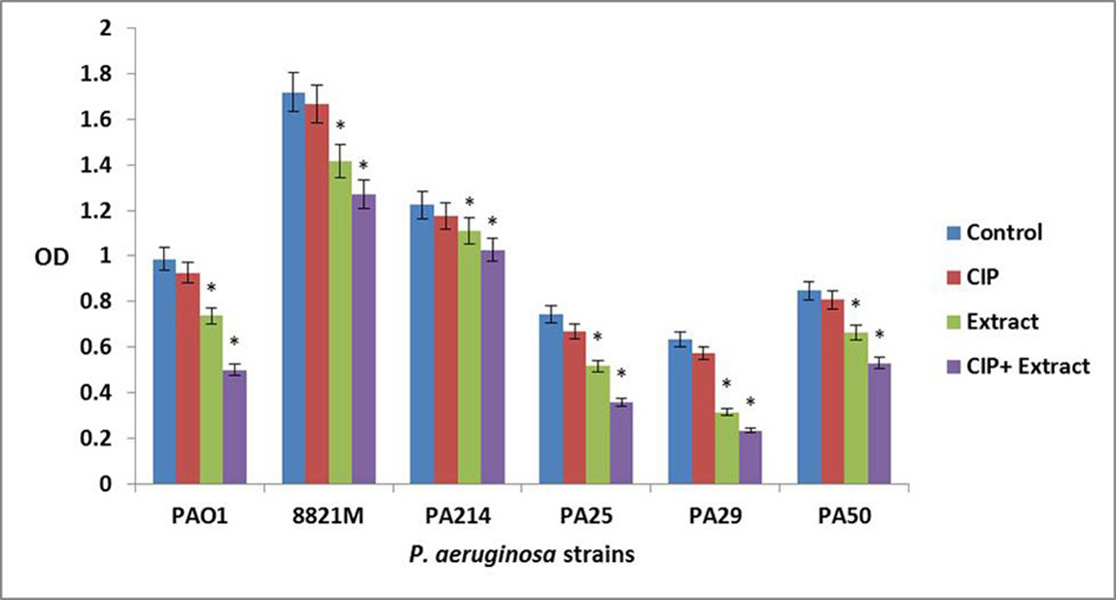
The Effect of Ciprofloxacin (CIP), *n*-butanolic* C. coum* Extract (Extract) and the Combination of Ciprofloxacin and *n*-butanolic *C. coum* Extract (CIP+Extract) on three day old Biofilm of six *P. aeruginosa* Strains Bars represent the mean OD570 values and error bars represent standard deviations. Blue bars, control samples treated with TSB medium alone; red bars, samples treated with ciprofloxacin (CIP); green bars represent biofilms treated with *n*-butanolic *C. coum* extract (Extract); purple bars represent biofilms treated with the combination of ciprofloxacin and n-butanolic *C. coum *extract (CIP+Extract). Statistical analysis was done using Student’s t-test and the P value < 0.05 was considered as significant (noted with *).

The ciprofloxacin did not affect three day old biofilm mass at all concentrations. The *n*-butanolic *C. coum* extract at 140 µg/mL eliminated three day old *P. aeruginosa* biofilms. However, *n*-butanolic *C. coum* extract (at 95 µg/mL) in combination with ciprofloxacin (at 4×MIC) dramatically dispersed three day old preestablished biofilm (Figure 3). The ciprofloxacin, *n*-butanolic *C. coum* extract and their combination did not significantly affect five day old biofilm mass (data not shown). By checkerboard synergy technique, *n*-butanolic *C. coum* extract in combination with ciprofloxacin exhibited synergistic effect against one and three day old *P. aeruginosa* biofilms (ΣFBEC ≤ 0.4) (9).

**Table 1.** Minimum Inhibitory Concentration (MIC) of *n*-butanolic *C. coum* Extract and Ciprofloxacin Using Agar Well Diffusion and Microtiter Plate Methods According to the CLSI Protocols.

*P. aeruginosa *Strains	*n*-butanolic *C. coum* extract	Ciprofloxacin	
	MIC, µg/mL	MIC, µg/mL	MBC ^a^, µg/mL
**PAO1**	256	0.25	0.5
**8821M**	256	1	2
**214**	254	4	8
**25**	254	8	16
**29**	257	8	16
**50**	257	4	8

^a^Minimum Bactericidal Concentration

## 5. Discussion

The emergence of infections due to highly resistant *P. aeruginosa, *which is capable of forming biofilms, causes a significant problem in the medical field, today. The healthcare community is not able to effectively treat such infections with the old classes of antibiotics, which rapidly lose their efficacy. Effective treatments for the elimination of established biofilms could diminish the morbidity and subsequently reduce healthcare costs associated with nosocomial infection (1).

In recent years, the molecules that control bacterial biofilms through nonmicrobicidal mechanisms have gained a lot of utility and attention. These molecules can act synergistically with conventional antibiotics to overcome infectious diseases (1). The antibiofilm agents will disperse bacterial biofilms, while the antibiotics will eliminate the bacterial population. Therefore, it is essential to determine how conventional antibiotics affect the ability of antibiofilm agents to control biofilm perpetuation (2).

For this reason, we studied the ability of *n*-butanolic *C. coum* extract to disperse bacterial biofilms in the presence of an approved antibiotic such as ciprofloxacin. At first, biofilm-forming ability of six different *P. aeruginosa* strains was investigated using microtiter plate and PCR (for the detection of adhesion genes) methods. All tested strains were able to form biofilms (Figure 1). According to Vallet et al. the CupA system plays a fundamental role in biofilm formation. They confirmed that the CupA-dependent adhesions are more required at an earlier stage of biofilm formation than type IV pili (10).

To quantify any synergistic effects between *n*-butanolic *C. coum* extract and ciprofloxacin toward eliminating biofilm colonization, pre-formed *P. aeruginosa* biofilms were treated with the combination of *n*-butanolic *C. coum* extract and ciprofloxacin. In this study, ciprofloxacin was investigated because it is widely prescribed to treat *P. aeruginosa* infections. In the current study, the combination of ciprofloxacin and plant extract showed a drastic effect towards dispersion of one and three day old *P. aeruginosa* biofilms (P ˂ 0.05). A 2-fold drop and a 4-fold drop in MIC were noted with ciprofloxacin, when one and three day old biofilms were pretreated with *n*-butanolic *C. coum* extract. Therefore, high levels of synergy were observed when low concentrations of *n*-butanolic *C. coum* were used in combination with the antibiotic. However, compared with the samples treated with medium only, this combination did not affect the five day old biofilm mass (in all concentrations examined). It seems that this phenomenon is due to the overproduction of alginate and over thickening of the biofilm mass.

The exact mechanism of synergy is currently unknown and further work is required to reveal the role and effect of cellular and molecular parameters on biofilm dispersion to extend the benefits of this synergy. Based on the observation that *n*-butanolic *C. coum* extract contains high levels of saponins, it is reasonable to assume the extract may re sensitize resistant bacteria to the antibiotics by inhibiting cell-to-cell communication (Quorum sensing). It has been revealed that quorum sensing has significant role in biofilm formation and the production of virulence factors. Quorum-sensing systems are important targets to address the sensitivity of bacteria to antibiotic compounds and the host immune system (11). By using *n*-butanolic *C. coum* extract, quorum sensing was inhibited and eventually the biofilm was dispersed and bacterial population was eliminated by ciprofloxacin. While these results are based on the analysis of crude, unfractionated extracts, they are also the first of many steps towards the development of new drugs with biofilm dispersal property.

In conclusion, it was demonstrated that the combination of an antibiofilm agent containing saponin compounds with conventional antibiotics provides an effective strategy for remediating biofilm based diseases.
